# Three bilberry bHLHs of subgroup IIIf play divergent roles in fruit anthocyanin and flavonoid biosynthesis

**DOI:** 10.1038/s41598-025-15557-w

**Published:** 2025-08-14

**Authors:** Katja Karppinen, Lauri Raami, Hilary Edema, Muhammad Furqan Ashraf, Heikki M. Salo, Richard V. Espley, Laura Jaakola

**Affiliations:** 1 Department of Arctic and Marine Biology, UiT The Arctic University of Norway, Tromsø, Norway; 2 Ecology and Genetics Research Unit, University of Oulu, Oulu, Finland; 3 Plant and Food Research Group, Bioeconomy Science Institute, Auckland, New Zealand; 4 Division of Food Production and Society, Norwegian Institute of Bioeconomy Research (NIBIO), Ås, Norway

**Keywords:** Anthocyanin biosynthesis, Basic helix-loop-helix transcription factor, Berry ripening, MBW regulatory complex, *Vaccinium myrtillus*, Plant development, Plant hormones, Plant molecular biology, Secondary metabolism

## Abstract

Bilberry (*Vaccinium myrtillus* L.) fruit are one of the best natural sources of anthocyanins. Anthocyanin and flavonoid biosynthesis are transcriptionally regulated by the conserved MBW complex, including R2R3 MYB, basic helix-loop-helix (bHLH) and WD40 proteins. In this study, homology-search in the bilberry genome identified three putative anthocyanin biosynthesis-regulating *bHLH* genes, all containing a MYB binding domain, indicating their possible interaction with R2R3 MYBs. Phylogenetic analysis assigned the VmbHLHs to three different clusters within the IIIf subgroup of bHLHs. The expression of *VmbHLH2* elevated at berry ripening, while *VmbHLH1* and *VmbHLH3* showed decreasing expression towards berry maturation. When transiently overexpressed together with *MdMYB10* in *Nicotiana benthamiana* leaves, *VmbHLH2* and *VmbHLH3* were able to complement *MdbHLH3* and upregulate a full set of anthocyanin biosynthetic genes leading to anthocyanin accumulation. In contrast, *VmbHLH1* induced a reduced set of flavonoid pathway genes. Our results provide the first functional characterization of flavonoid-regulating bHLHs that contribute to the MBW complex in the economically important genus *Vaccinium.* We propose that the TT8-type VmbHLH2 is involved in anthocyanin biosynthesis in ripening bilberry fruit, while the MYC1-type VmbHLH1 and GL3/EGL3-type VmbHLH3 are associated with anthocyanin and PA biosynthesis, mainly in tissues other than berries.

**Supplementary Information:**

The online version contains supplementary material available at 10.1038/s41598-025-15557-w.

## Introduction

Basic helix-loop-helix (bHLH) transcription factors (TFs) comprise a large gene family in plants, with over 160 members identified in Arabidopsis and more than 180 in rice^1^. As the second largest TF family in plants, bHLHs are involved in many growth and developmental processes, including regulation of hormone signaling, responses to biotic and abiotic stresses and regulation of flavonoid and anthocyanin biosynthesis^2^. The bHLH proteins can activate or repress gene expression through binding DNA promoter regions, in the form of bHLH homo- or heterodimers, or in a complex associated with proteins from other TF families, such as MYBs^3^.

Anthocyanins contribute to the red and blue colors in many flowers and ripe fruits, such as berries, facilitating pollination and seed dispersal^4^. Many fruits and berries are recognized as rich sources of anthocyanins and are associated with various health benefits^5–8^. Anthocyanins are produced from the well-elucidated flavonoid biosynthetic pathway which consists of several consecutive enzymatic steps leading to different flavonoid classes, namely anthocyanins, proanthocyanidins (PAs) and flavonols^9^. The transcription of flavonoid biosynthetic structural genes encoding these enzymes is controlled by the conserved MBW regulatory complex, consisting of R2R3 *M*YB, *b*HLH and *W*D40 proteins^10–12^. In this complex, the bHLH physically interacts with a MYB partner to enable activation or repression of target genes. The expression pattern and the DNA-binding specificity of MYBs and bHLHs determine the activation of flavonoid pathway target genes in plants^13,14^. Flavonoid regulation is considered to be the most ancient function of the MBW complex, pre-dating MBW roles in development of trichomes and root hairs and production of seed coat mucilage, which evolved after gene duplication and subsequent diversification^10^.

Within the bHLH family, several members of subgroup IIIf have been associated with anthocyanin and flavonoid biosynthesis. In most species, more than one bHLH is generally found to operate within MBW complexes to regulate anthocyanin and PA biosynthesis^3,15^. For example, in Arabidopsis, four members of the subgroup IIIf bHLHs, transparent testa 8 (TT8; AtbHLH042), AtMYC1 (AtbHLH012), glabra3 (GL3; AtbHLH001) and enhancer of glabra3 (EGL3; AtbHLH002), have all been associated with the control of anthocyanin and PA biosynthesis, in a partially redundant manner^3,16,17^. In Petunia (*Petunia hybrida*), two well-characterized bHLHs, PhAN1 and PhJAF13, regulate anthocyanin biosynthesis in floral organs together with the R2R3 MYB partner, PhAN2, although PhAN1 also assists in controlling vacuolar pH with another R2R3 MYB, PhPH4^18,19^. Similarly, two divergent bHLHs in snapdragon (*Antirrhinum majus*), AmDELILA and AmIncolorata I/Mutabilis, play partly overlapping roles with AmROSEA1 in anthocyanin-derived flower color^20,21^.

Several members of subgroup IIIf bHLHs have also been identified in fruit and berry flavonoid biosynthesis. Two bHLHs exist in grape (*Vitis vinifera*), of which VvMYCA1 interacts with VvMYBA2 to induce anthocyanin biosynthesis, while VvMYC1 interacts with several R2R3 MYBs, to control both anthocyanin and PA biosynthesis in berry skin^22,23^. Two homologous bHLH proteins in apple (*Malus domestica*), MdbHLH3 and MdbHLH33, regulate anthocyanin biosynthesis together with MdMYB10, allelic to MdMYB1, in the MBW complex^24–26^. MdbHLH3 binds the promoters of anthocyanin biosynthesis genes *MdDFR* and *MdUFGT* to regulate their expression^27^. Two bHLH proteins also exist in Chinese bayberry (*Morella rubra*), of which MrbHLH1, but not MrbHLH2, is an essential partner for MrMYB1 in berry anthocyanin production^28^. In black goji berry (*Lycium ruthenicum* Murray), either LrAN1b or LrJAF13 form MBW complex with R2R3 MYB and WD40 protein in regulating anthocyanin biosynthesis^29^. In white goji berry variant, a deletion in the *LrAN1b* promoter region abolishes expression of the *LrAN1b*, leading to the absence of pigmentation^29^. In woodland strawberry (*Fragaria vesca*), three distinct bHLHs have been demonstrated to interact with several R2R3 MYBs in regulating expression of flavonoid structural genes^30^. The orchestration of these key factors in coordinating different branches of flavonoid biosynthesis is not completely understood across all fruit species, including berries which often show more complex anthocyanin and PA profiles.

To deepen our understanding of the regulatory role of MBW-associated bHLHs, we focused on the identification of anthocyanin-regulating bHLHs in bilberry (*Vaccinium myrtillus* L.), which has active flavonoid metabolism over the course of fruit development and ripening. While astringent PAs mainly accumulate in bilberry fruit in the early stages of development, anthocyanins accumulate at high concentrations in ripe fruit^31–33 ^and make bilberry one of the best sources of anthocyanins. In bilberry, the ripening-related anthocyanin biosynthesis is positively regulated by the phytohormone abscisic acid (ABA)^34,35^. ABA has a well-known regulatory role in promoting fleshy fruit maturation through many ripening-related processes, including accumulation of anthocyanin pigments by upregulating the expression of key biosynthetic genes and TFs regulating them^36^. Fleshy fruit ripening is a tightly regulated developmental process. Especially in non-climacteric fruit, but also in climacteric fruit, ABA is acting as a ripening inducer by interacting with other plant hormones, including ethylene, auxin, gibberellic acid and brassinosteroids^37,38^.

Recently, we provided a comprehensive report of bilberry flavonoid-related R2R3 MYBs, showing that VmMYBA1, VmMYBPA1.1, and VmMYBPA2.2 co-regulate ripening-related anthocyanin biosynthesis in bilberry fruit^39^. However, the bHLHs associated with anthocyanin biosynthesis as part of the MBW complex have not yet been verified in bilberry, or the genus *Vaccinium*, which contains commercially important species, such as highbush blueberry (*V. corymbosum*) and American cranberry (*V. macrocarpon*). In highbush blueberry, over 50 *bHLH* family members have been identified^40,41^. Gene expression patterns indicate that some of these bHLHs could be associated with flavonoid biosynthesis^42–46^. However, the functional characterization of bHLH regulators within the MBW complex is still lacking in the genus *Vaccinium*. Since bHLHs are integral parts of the MBW complexes and protein-protein interactions with bHLHs are known to affect the DNA-binding properties of the R2R3 MYB components^30^, identification of bHLH partners is important for further elucidation of the flavonoid and anthocyanin biosynthesis regulation in genus *Vaccinium*.

In this study, three potential anthocyanin-regulating *bHLH* genes were identified in the bilberry genome of the Norwegian ecotype^47^. Their role in anthocyanin biosynthesis was studied by gene expression analysis in bilberry, after ABA treatment of berries, and by overexpression analyses in *Nicotiana benthamiana* leaves. VmbHLH2 and VmbHLH3 were found to regulate the complete anthocyanin biosynthesis pathway leading to anthocyanin accumulation. Further, VmbHLH1 induced specific genes in the flavonoid pathway. Our results suggest that these three bHLHs act as a part of the flavonoid-regulating MBW complex and exhibit divergent and partially overlapping roles in anthocyanin biosynthesis in bilberry tissues.

## Results

### Bilberry subgroup IIIf *bHLH* genes

To study the regulation of anthocyanin biosynthesis in bilberry by bHLH TFs, three putative anthocyanin-regulating *bHLHs* were identified in the bilberry genome based on sequence homology with Arabidopsis subgroup IIIf bHLH members (Table 1). When the three genes were cloned and fully sequenced, they showed some size variation: *VmbHLH1* was 1923 bp, *VmbHLH3* 1803 bp, while *VmbHLH2* was 2190 bp long. The nucleotide sequences clearly represented separate genes with *VmbHLH1* showing 54.42% similarity to *VmbHLH2* and 65.06% similarity to *VmbHLH3*, while *VmbHLH2* and *VmbHLH3* shared 53.29% identity (Table 1). When phylogenetic analysis was performed with 169 Arabidopsis bHLHs, the three VmbHLHs were clustered with known flavonoid biosynthesis-regulating AtbHLHs belonging to the subgroup IIIf described by Heim et al.^15, ^namely AtbHLH001, AtbHLH002, AtbHLH012 and AtbHLH042 (Fig. S1).

**Table 1:** Characteristics of the sequences of Bilberry *bHLH* genes.

Gene	Accession ID^1^	Characteristics			Sequence identity at nucleotide level (%) ^2^					
		CDS (bp)	Amino acids	Subgroup	*VmbHLH1*		*VmbHLH2*		*VmbHLH3*	
*VmbHLH1*	Vmy12g484.t1	1923	640	IIIf	100		54.42		65.06	
*VmbHLH2*	Vmy06g33670.t1	2190	729	IIIf			100		53.29	
*VmbHLH3*	Vmy10g9347.t1	1803	600	IIIf					100	

^1^Retrieved from the Genome Database for *Vaccinium* (https://www.vaccinium.org).

^2^The values were obtained from sequence alignments on Clustal Omega. CDS, coding sequence; bp, base pair.

### The bilberry bHLHs show sequence similarity to known flavonoid biosynthesis regulators

**Fig. 1:**
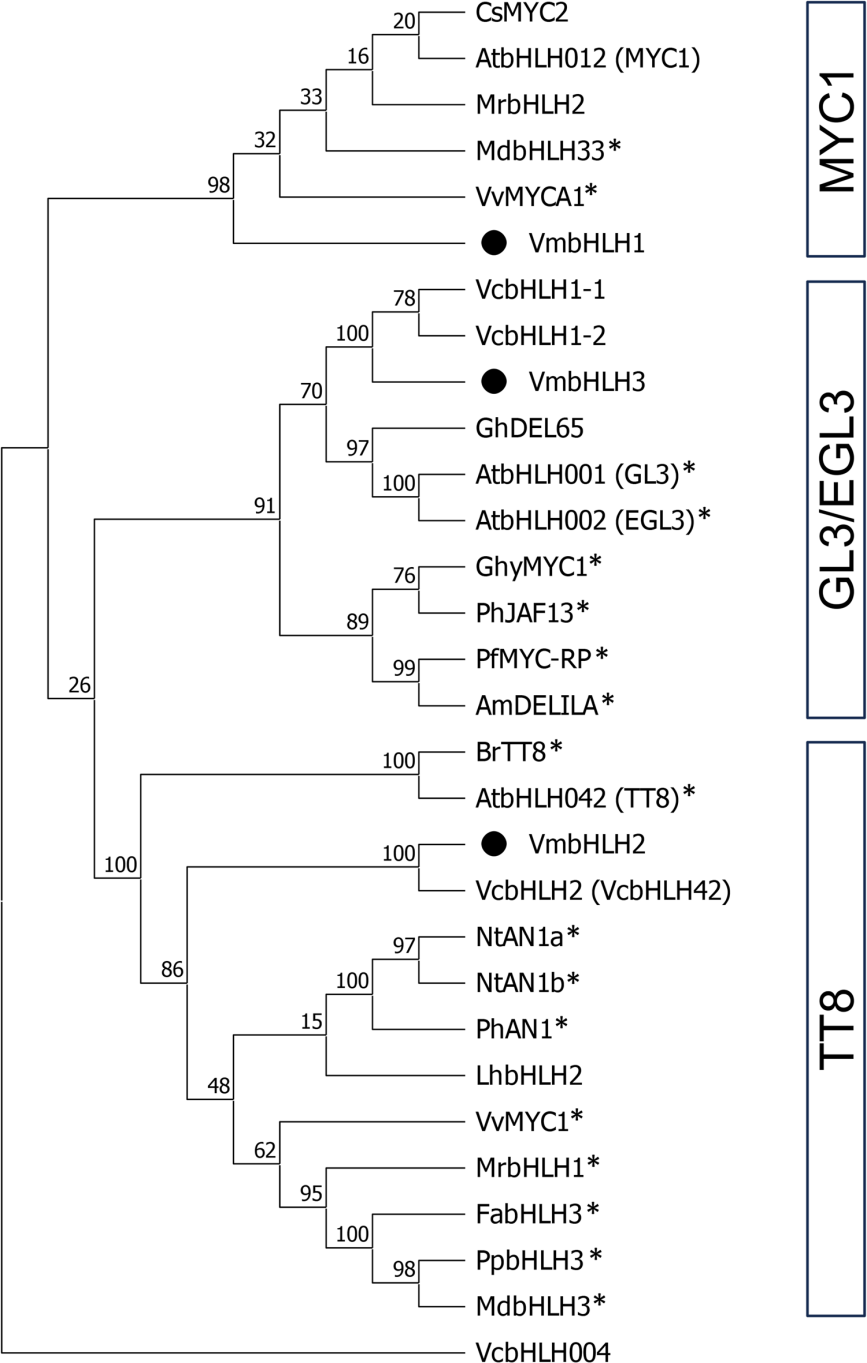
Phylogenetic analysis of subgroup IIIf bHLHs. The bilberry bHLHs are indicated as black circles. Asterisks indicate functionally characterized flavonoid biosynthesis-regulating IIIf bHLHs in other plant species. The tree was constructed using MEGA 11 and numbers near branches indicate bootstrap values in percentage (1000 replicates). Am, *Antirrhinum majus;* At, *Arabidopsis thaliana*; Br, *Brassica rapa;* Cs, *Citrus sinensis;* Fa, *Fragaria x ananassa*; Gh, *Gossypium hirsutum;* Ghy, *Gerbera hybrida;* Lh, *Lilium hybrid;* Md, *Malus domestica*; Mr, *Morella rubra;* Nt, *Nicotiana tabacum;* Pf, *Perilla frutescens;* Ph, *Petunia x hybrida;* Pp, *Prunus persica*; Vc, *Vaccinium corymbosum*; Vm, *Vaccinium myrtillus*; Vv, *Vitis vinifera*.

A closer phylogenetic examination with the subgroup IIIf of flavonoid biosynthesis-regulating bHLHs showed three major clades which were strongly supported by bootstrap values ≥ 91% (Fig. 1). The three bilberry bHLHs were clustered in different clades. VmbHLH1 grouped among MYC1-type bHLHs (bootstrap support 98%), which included grapevine VvMYCA1, apple MdbHLH33 and MrbHLH2 from Chinese bayberry, and showed 48.58% identity with Arabidopsis AtbHLH012 (MYC1). VmbHLH2 clustered in a large clade containing previously characterized TT8-type bHLHs in anthocyanin/PA biosynthesis regulation (bootstrap support 100%), including MdbHLH3, MrbHLH1, VvMYC1, PpbHLH3 and NtAN1a/b, and also showed 57.88% identity to AtbHLH042 (TT8). VmbHLH2 appears to be a homolog of the blueberry VcbHLH2 (VcbHLH042)^42–46^ with 98.35% similarity. Conversely, VmbHLH3 grouped with previously characterized GL3/EGL3-type bHLHs associated with anthocyanin biosynthesis regulation (bootstrap support 91%), including Antirrhinum AmDELILA and Gerbera GhyMYC1, and with 52.74% identity to AtbHLH001 (GL3) and 53.18% to AtbHLH002 (EGL3) (Fig. 1). VmbHLH3 seems to be homologous to blueberry VcbHLH1-1 and VcbHLH1-2 proteins^44^, with 97.00% and 96.67% similarity, respectively. The previously described blueberry VcbHLH004^48^ showed no sequence homology with IIIf subgroup members and lacked the MYB interaction region.

**Fig. 2:**
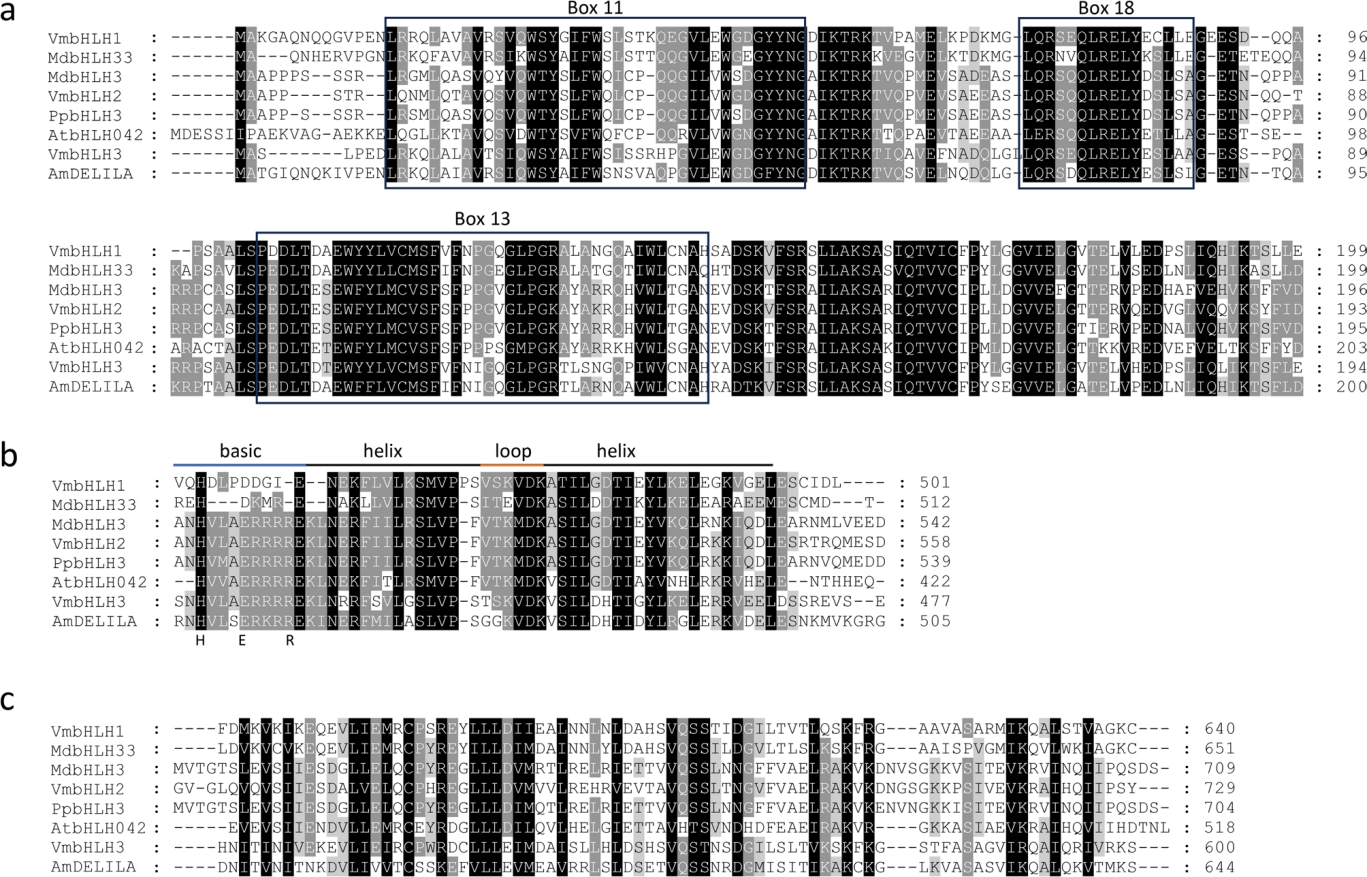
Identification of conserved domains in bilberry bHLHs. Amino acid sequence alignment of three VmbHLH proteins and the known anthocyanin bHLH regulators in other species indicate presence of (A) MYB interaction region, (B) bHLH domain and (C) ACT-like domain. Conserved residues are highlighted in black, and partial conservation is indicated in grey. Am, *Antirrhinum majus;* At, *Arabidopsis thaliana*; Md, *Malus domestica*; Pp, *Prunus persica*; Vm, *Vaccinium myrtillus.*

Alignment of the VmbHLH amino acid sequences with well-characterized subgroup IIIf bHLHs in anthocyanin biosynthesis showed a strong sequence similarity and the presence of three conserved regions (Fig. 2). The N-terminus of all three VmbHLHs contained the MYB interaction region, including the characteristic boxes 11, 13 and 18 **(**Fig. 2A**)** conserved within the IIIf subgroup, which enable the binding and interaction with suitable MYB partners in the MBW complex^15,24,49^. All VmbHLH sequences also contained the signature basic helix-loop-helix (bHLH) domain although only VmbHLH2 and VmbHLH3 showed a conserved HER (His5-Glu9-Arg13) motif (Fig. 2B), which is found in anthocyanin biosynthesis-regulating bHLH TFs. All three VmbHLHs contained the C-terminal transactivation (ACT) domain (Fig. 2C) which enables transcription^3,49^.

### *VmbHLHs* show differential expression patterns in bilberry

**Fig. 3:**
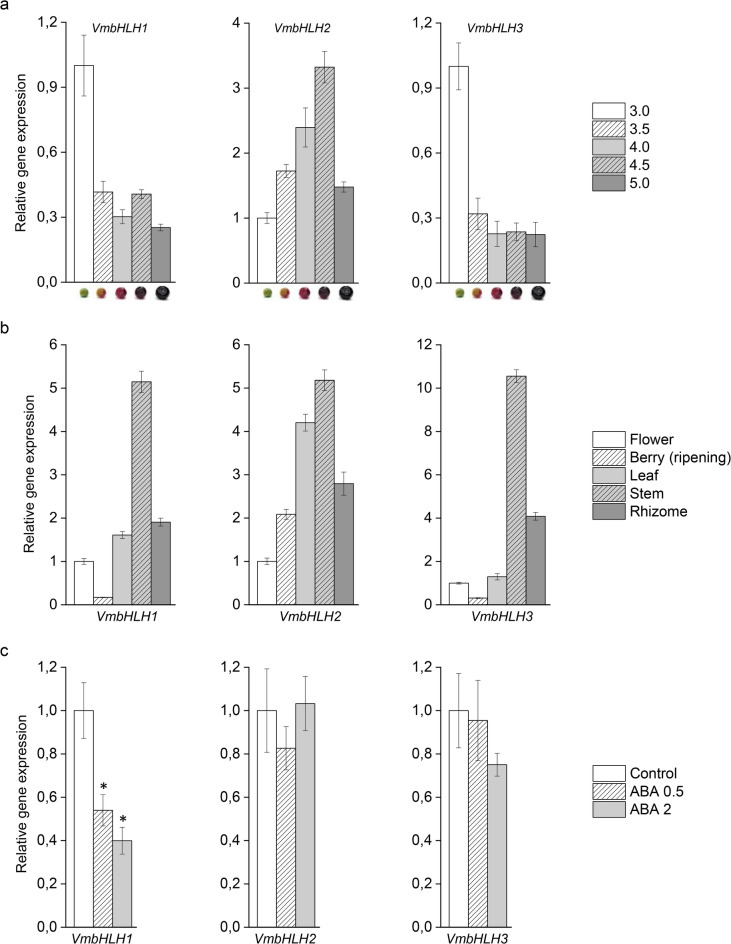
The expression profiles of *VmbHLH* genes (A) during bilberry fruit ripening, (B) in various bilberry tissues and (C) after 48-h treatment of unripe fruit by 0.5 mM ABA, 2 mM ABA or water (control). The relative gene expression was quantified by qPCR and normalized to *VmGAPDH* and *VmActin*. Values represent means ± SEs of four replicates. Asterisks indicate significant differences from control in Student’s *t*-test (*p* ≤ 0.05). 3.0–5.0 represent fruit ripening stages from unripe to fully ripe berry.

To investigate the spatial and temporal expression patterns of the *VmbHLH* genes, transcript abundance was measured during berry ripening and in various bilberry tissues, including flower, ripening berry, leaf, stem and rhizome. Our real-time quantitative PCR (qPCR) analyses demonstrated the transcript levels of *VmbHLH1* and *VmbHLH3* to be highest in the unripe berry followed by a decrease in the later berry-ripening stages (Fig. 3A). Conversely, the expression of *VmbHLH2* was upregulated during berry ripening and expression peaked at stage 4.5, just before berries were fully ripe (Fig. 3A). This expression pattern of *VmbHLH2* follows anthocyanin accumulation^32^ and resembles the expression patterns of the key bilberry anthocyanin biosynthesis genes and R2R3 MYB regulators during bilberry fruit ripening (Fig. S2).

For all three *VmbHLH* genes, expression was found to be highest in the bilberry stem, although *VmbHLH2* transcripts were also detected at relatively high levels in leaves, followed by rhizome, ripening berries and flowers (Fig. 3B), indicating roles in both reproductive and vegetative tissues. In contrast, the expression of *VmbHLH1* and *VmbHLH3* was relatively low in reproductive tissues, especially berries, compared with those in vegetative tissues.

Since bilberry fruit ripening is mediated by ABA^35^, we applied ABA treatment to unripe berries to test its impact on the expression of *VmbHLHs*. Both the 0.5 mM and 2 mM ABA treatments significantly downregulated *VmbHLH1* expression (Fig. 3C). Only slight concentration-dependent downregulation was observed with *VmbHLH3*, while *VmbHLH2* expression was unaffected by ABA (Fig. 3C), indicating that *VmbHLH2* expression is not regulated by the ABA signal.

### *VmbHLH2* and *VmbHLH3* induce anthocyanin accumulation when co-expressed with *MdMYB10*

**Fig. 4:**
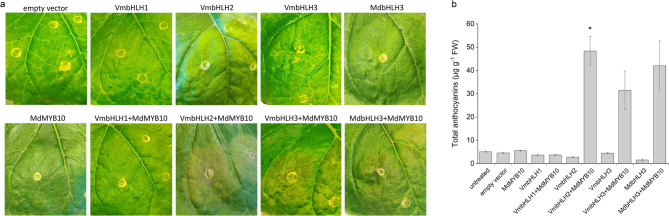
Effect of transient overexpression of *VmbHLHs* on anthocyanin accumulation in *Nicotiana benthamiana* leaves. (A) Pigmentation in *N. benthamiana* leaves after 7 days of infiltration. Infiltration of *bHLHs* was conducted either alone or with *MdMYB10.* (B) Total anthocyanin content in *N. benthamiana* leaves after 7 days of infiltration. Untreated leaves, infiltration with empty vector and *VmbHLH* or *MdMYB10* alone served as negative controls. Values represent means ± SEs of at least three biological replicates. Asterisks indicate significant differences in anthocyanin concentration from infiltration with *MdMYB10* alone in Student’s *t*-test (*p* ≤ 0.05). FW, fresh weight.

To functionally characterize bilberry bHLHs, *Agrobacterium*-mediated transient overexpression assays in *N. benthamiana* leaves were performed. The *VmbHLHs* were separately co-expressed with anthocyanin biosynthesis-regulating apple *MdMYB10*, which is known to be dependent on the co-expression of a suitable bHLH partner in *Nicotiana* leaves, such as MdbHLH3^24,50^. This provides an excellent system for functional characterization of ectopic bHLHs since many A-type bHLHs, including VmMYBA1 and VmMYBA2 of bilberry^39^, do not require the co-expression of a bHLH for anthocyanin accumulation in *Nicotiana* leaves. Anthocyanins were detected in *N. benthamiana* leaves after seven days following infiltration with either *VmbHLH2* or *VmbHLH3* or *MdbHLH3* (positive control) together with *MdMYB10* (Fig. 4A). Pigmentation was most visible with *VmbHLH2*. No pigmentation was observed when leaves were infiltrated either with *VmbHLH1* + *MdMYB10* or with any of the *VmbHLHs* alone, or *MdMYB10* alone, or with negative controls (empty vector or untreated) (Fig. 4A). The leaf infiltration sites were confirmed for the presence of *VmbHLH* expression (Fig. S3).

Anthocyanin content analysis at infiltration sites confirmed that *VmbHLH2* + *MdMYB10* induced the highest concentration of anthocyanin while *VmbHLH3* together with *MdMYB10* induced slightly lower amounts than *VmbHLH2* + *MdMYB10* or *MdbHLH3* + *MdMYB10* (Fig. 4B). Anthocyanin in *Nicotiana* leaves infiltrated either with *VmbHLH1* + *MdMYB10*, *VmbHLHs* alone, or *MdMYB10* alone, was in similar concentration to those with the empty vector or untreated controls (Fig. 4B).

### VmbHLHs activate the expression of specific sets of anthocyanin biosynthetic genes

**Fig. 5:**
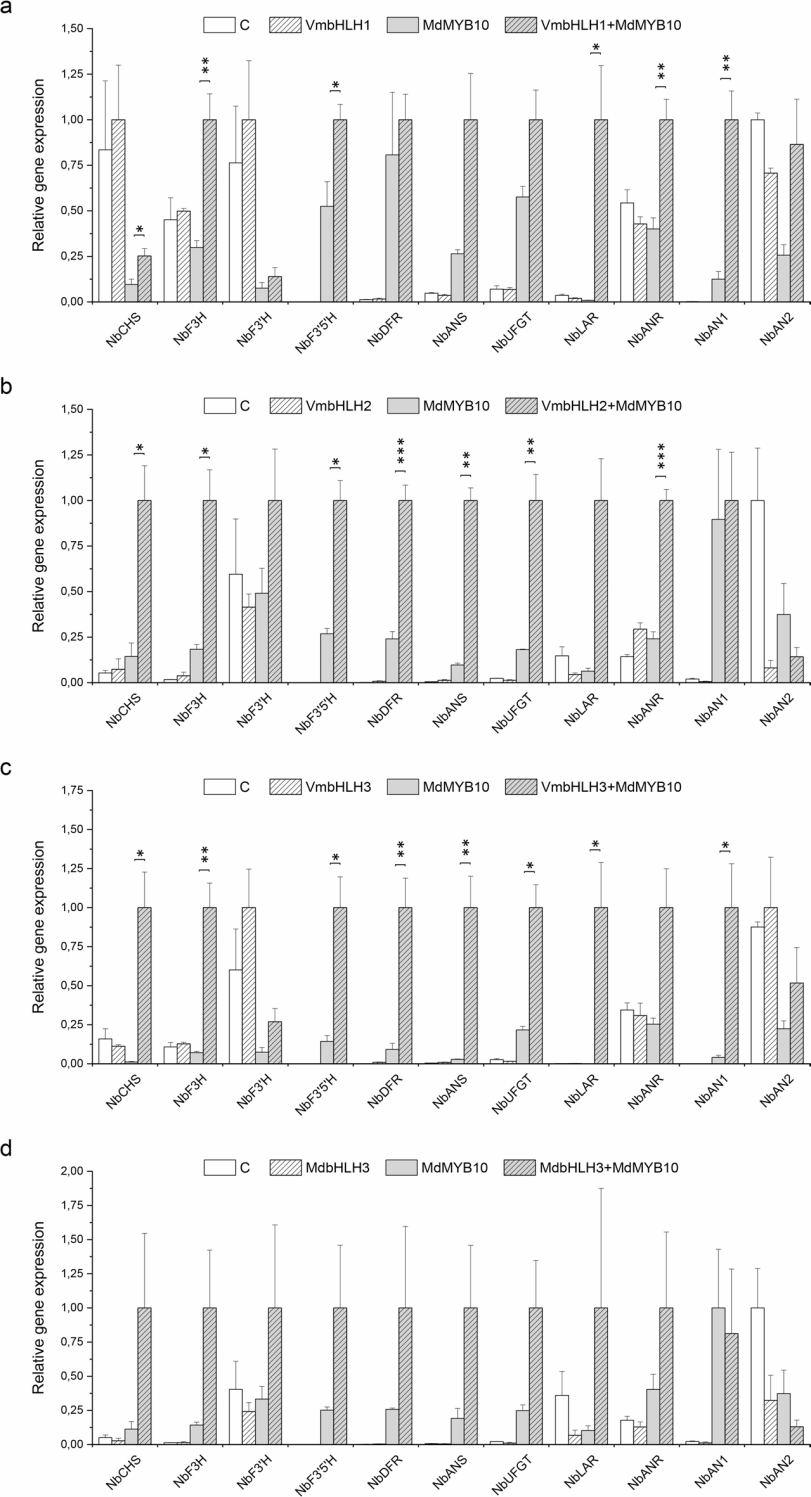
Expression of flavonoid biosynthetic genes in *Nicotiana benthamiana* leaves after transient overexpression with (A) *VmbHLH1*, (B) *VmbHLH2*, (C) *VmbHLH3* and (D) *MdbHLH3* (positive control). Relative expression of the genes was quantified from infiltration sites after 7 days of infiltration by qPCR and normalized to *NbEF1* and *NbActin*. Values represent means ± SEs of at least three biological replicates. Asterisks indicate significant differences from infiltration with *MdMYB10* alone according to Student’s *t*-test (*, *p* ≤ 0.05; **, *p* ≤ 0.01; ***, *p* ≤ 0.001). C, empty vector control.

The regulatory role of *VmbHLHs* in anthocyanin biosynthesis was further analyzed for flavonoid biosynthetic structural gene expression. Results showed that, although *VmbHLH1* did not induce accumulation of anthocyanins in *N. benthamiana* leaves when co-expressed with *MdMYB10*, it could upregulate a restricted set of structural genes in the flavonoid pathway. Significant upregulation of *NbF3H*,* NbF3’5’H*, *NbLAR* and *NbANR* expression was detected, while the expression of *NbANS* and *NbUFGT* was also slightly enhanced when compared with infiltration with *MdMYB10* alone (Fig. 5A**)**. *VmbHLH2*, together with *MdMYB10*, significantly upregulated almost all the tested flavonoid and anthocyanin biosynthetic genes, while *NbF3’H* expression was only slightly increased (Fig. 5B). This indicates that it can complement *MdbHLH3* and induce a similar expression pattern among anthocyanin and flavonoid biosynthetic genes (Fig. 5D). *VmbHLH3* also activated almost all the anthocyanin and flavonoid biosynthetic genes when co-expressed with *MdMYB10*, the exception of *NbF3’H* (Fig. 5C). In addition, *VmbHLH1* and *VmbHLH3* upregulated the endogenous bHLH *NbAN1*. The expression of *NbFLS* was unchanged by *bHLH* infiltration (Fig. S4).

## Discussion

We previously conducted a comprehensive study of bilberry flavonoid-regulating R2R3 MYB TFs^39^, but their bHLH partners interacting in the MBW complex had not been functionally characterized in *Vaccinium* berries before the present study. In earlier reports, *VcbHLH2* and *VmbHLH2* sequences were found to be unstable in plasmids, possibly because of a microsatellite repeat, restricting functional analyses^45,51^. The lack of bHLH partners poses a limitation to studies on the regulation by the MBW complexes and their regulatory targets in flavonoid biosynthesis in *Vaccinium* berries. Here, we identified, cloned and functionally characterized three *bHLH* genes from bilberry which, based on our results, are associated with anthocyanin and flavonoid biosynthesis.

All three VmbHLHs showed high similarity to bHLHs of subgroup IIIf of Arabidopsis and other plant species, members of which are known to regulate flavonoid biosynthesis^3^. Phylogenetic analysis showed that the bilberry bHLHs were separated under three clades in the IIIf subgroup, which have been described previously by Starkevič et al.^52^.

The VmbHLH1 sequence was clustered with MYC1-type bHLHs, including the anthocyanin biosynthesis-regulating apple MdbHLH33 and grapevine VvMYCA1^23,24^. VmbHLH2 was found to be homologous to TT8-type bHLHs, which include MdbHLH3, MrbHLH1, VvMYC1, PpbHLH3, PhAN1 and AtTT8, all of which have been reported to function in anthocyanin and PA biosynthesis regulation^18,22,24,27,28,53,54^. For example, Arabidopsis AtTT8 is involved in the regulation of both anthocyanin and PA biosynthesis in seeds and seedlings^55–57^. Our results further indicated that VmbHLH3 shared identity with GL3/EGL3-type bHLHs. In our phylogenetic tree, VmbHLH3 clustered with Antirrhinum AmDELILA, Petunia PhJAF13 and Gerbera GhyMYC1, as well as Arabidopsis AtGL3 and AtEGL3, which are associated with the regulation of anthocyanin biosynthesis in flower petals and roots, respectively^16–18,56,58,59^. Several studies have demonstrated that AmDELILA functions together with the R2R3 MYB, AmROSEA1, for the production of high anthocyanin concentration in different overexpression systems^60–62^.

The role of conserved MBW complexes in regulating anthocyanin and PA biosynthesis is relatively well understood. The MYB interaction region in the bHLH sequence is generally considered indispensable for the interaction with the R2R3 MYBs that regulate anthocyanin and PA biosynthesis^49,63^. All three VmbHLHs contained an intact MYB interaction area in their N-terminus, indicating a possible protein-protein interaction with R2R3 MYBs. In these complexes, the R2R3 MYB interacts with a bHLH to induce anthocyanin/flavonoid accumulation by activating promoters of flavonoid structural genes. Apple MdMYB10 (an allele of MdMYB1) is an example of a MYBA-type R2R3 MYB, which is dependent on the presence of a suitable bHLH partner, such as *MdbHLH3*,* MdbHLH33* or *AtbHLH002*, for the induction of anthocyanin biosynthesis genes when overexpressed in *Nicotiana* leaves^24,50,64^. Similarly, in our study, *MdMYB10* alone was not capable of inducing anthocyanin accumulation in *N. benthamiana* leaves, although it upregulated the expression of a restricted set of flavonoid structural genes. However, this was greatly enhanced when co-expressed with either *MdbHLH3*, *VmbHLH2* or *VmbHLH3* and led to anthocyanin accumulation. This indicates that the two bilberry bHLHs could co-operate with the MYBA-type MdMYB10 to induce a complete set of flavonoid and anthocyanin biosynthetic genes (except for *NbF3’H*). Our results also show that VmbHLH2 and VmbHLH3 can form MBW complexes with MdMYB10 in *N. benthamiana* leaves without addition of a bilberry WD40 protein construct.

It should also be noted that *MdbHLH3* and *VmbHLH2* induced similar expression patterns of the flavonoid structural genes, supporting the evidence for VmbHLH2 being orthologous to the TT8-type MdbHLH3. Conversely, GL3/EGL3-type VmbHLH3 induced slightly divergent expression patterns, particularly for *NbF3’H*, *NbLAR* and *NbAN1*, although it induced anthocyanin accumulation. The redundancy between bHLHs has been reported previously. In Arabidopsis, GL3/EGL3 can act redundantly with TT8 to control anthocyanin biosynthesis^56^. Also, in Antirrhinum GL3/EGL3-type AmDELILA has been shown to complement the loss of a TT8-type AmIncolorata I/Mutabilis function in anthocyanin biosynthesis^20,21^. In Petunia, both TT8-type AN1 and GL3/EGL3-type PhJAF13 are suggested in the regulation of anthocyanin biosynthesis but PhJAF13 does not compensate for the loss of AN1 in *an1* mutants and has been found to stimulate *PhAN1* expression during inductive conditions, such as fruit ripening, through a hierarchical mechanism^18,19,65^. The same mechanism has been reported in *Ipomoea* sp., pea and *Dahlia*^65^. The redundancy or hierarchical regulation between subgroup IIIf bHLHs have been studied less in berry anthocyanin biosynthesis. However, a recent study showed that LrJAF13 (PhJAF13 homolog) was not able to restore anthocyanin pigmentation in white goji berry variant caused by deletions in *LrAN1b* (PhAN1 homolog) promoter^29,66^. In fact, hierarchical regulation was found between two MBW complexes. LrJAF13-LrAN11(WD40)-LrAN2-like MBW complex upregulated *LrAN1b* expression by binding its promoter, leading to formation of LrAN1b-LrAN11-LrAN2-like MBW complex, which activated transcription of anthocyanin biosynthetic genes^29^.

*VmbHLH1* did not induce anthocyanin accumulation in *N. benthamiana* leaves but did induce the expression of structural genes of the flavonoid pathway, including *NbF3H*, *NbF3’5’H*,* NbANS*, *NbUFGT*, *NbLAR*, and *NbANR*. The inability to induce *NbCHS* and *NbDFR* expression is likely to explain the lack of anthocyanin accumulation in *N. benthamiana* leaves. This result is in accordance with the VmbHLH1 sequence lacking the conserved HER motif, which is known to control binding to bHLH-binding sites (E-box) in promoter regions of anthocyanin biosynthetic genes^3,67^. Our results are similar to that reported for Chinese bayberry bHLHs, as anthocyanin accumulation in tobacco leaves was demonstrated when anthocyanin-regulating *MrMYB1* was co-expressed with TT8-type *MrbHLH1*, but not when co-expressed with MYC1-type *MrbHLH2*^28^. On the other hand, grape VvMYCA1 was demonstrated to interact with VvMYBA2r to induce anthocyanin biosynthesis in tobacco leaves^23^. Thus, it seems that both MYC1-type and GL3/EGL3-type bHLHs of different species can demonstrate divergent functions and mechanisms in the regulation of flavonoid and anthocyanin pathway. This variability throughout the Eudicots is suggested to reflect functional specialization of the bHLH factor roles between genera^65^. The ability of VmbHLH1 and VmbHLH3 to attend in regulation of anthocyanin biosynthesis remains to be further explored in bilberry, which shows complex anthocyanin and flavonoid metabolism. None of the *VmbHLHs* induced expression of *NbFLS*, indicating that these VmbHLHs were not able to induce flavonol biosynthesis and their regulation is restricted to anthocyanin and PA pathway genes. This is in accordance with the current knowledge that flavonol biosynthesis-regulating MYBs (MYBF, SG7) act independently of bHLHs^49,63^.

When deciphering the role of *VmbHLHs* in bilberry, their spatial and temporal expression and that of their potential MYB partners should be considered. It has been shown that within the same species, MBW complexes can include different classes of MYBs and bHLHs to regulate different branches of the flavonoid pathway. For example, MYBA-type AtPAP1 or AtPAP2 together with AtTT8 and AtTTG1 regulate anthocyanin synthesis in Arabidopsis, whereas AtTT8 is also capable of acting with MYBPA2-type AtTT2 and AtTTG1 complexes to regulate PA biosynthesis via activation of BAN/ANR expression^55,57^. VvMYC1 has also been shown to interact with several R2R3 MYBs, such as VvMYB5a, VvMYB5b, VvMYBA1/A2 and VvMYBPA1, to control both anthocyanin and PA biosynthesis^22^. In our study, *VmbHLH1* expression was higher in unripe bilberry fruit than in ripening fruit and was significantly downregulated by ABA, the known inducer of fruit ripening and anthocyanin biosynthesis in bilberry^34,35^. These findings support our results from the overexpression experiments in that VmbHLH1 may not associate with anthocyanin biosynthesis regulation and indicate that its role in berry may be associated with PA biosynthesis, especially in the early stages of bilberry fruit development^33^. Our earlier study showed the expression of many of the PA-type R2R3 MYBs to be associated with early stages of bilberry fruit development and being downregulated by ABA^39^. A similar role was previously suggested by Günther et al.^43^, describing a partial sequence homolog of *VmbHLH1*, blueberry *VcbHLH1*, to be co-expressed with *VcMYBPA1* in a transcriptome of blueberry fruit flesh. This correlated with the accumulation of PAs and hydroxycinnamic acids. However, our study showed higher expression of *VmbHLH1* in bilberry tissues other than fruits, suggesting that the MYC1-type VmbHLH1 could also have a role in non-fruit tissues.

In contrast, the TT8-type *VmbHLH2* showed increased expression during bilberry fruit ripening. This coincided with its potential target flavonoid structural genes and potential A-type R2R3 MYB partner in anthocyanin biosynthesis, VmMYBA1. Earlier studies have also found the expression of *VmbHLH2* homologs to correlate with MYBA1 and MYBPA1.1 expression in fruit skin and anthocyanin biosynthetic genes^42,43,45,46^. Thus, our data suggests that *VmbHLH2* functions as a regulator of anthocyanin biosynthesis in ripening bilberry, probably in partnership with VmMYBA1. In contrast, although *VmbHLH3* could also complement *MdbHLH3* to induce anthocyanin accumulation in *N. benthamiana*, its expression in bilberry differs drastically from that of *VmbHLH2*, resembling that of *VmbHLH1* instead. Therefore, it is likely that *VmbHLH3* regulates PA biosynthesis in unripe berries and anthocyanin biosynthesis in tissues other than berries, which is supported by its apparent slight downregulation in response to ABA treatment. Anthocyanin regulation may be driven by interaction with the previously described VmMYBA2, which shows decreasing expression during fruit maturation and high expression in tissues other than fruits as well as being negatively regulated by ABA^39^. Xu et al.^30^ previously suggested a more extensive role for woodland strawberry GL3/EGL3-type bHLH than that of TT8- and MYC1-type bHLHs in flavonoid biosynthesis. The expression of this bHLH correlated with PA content and it was shown to interact with ten different R2R3 MYB TFs, including FvMYB10, and it was suggested to be a universal partner for FvMYB proteins^30^. The interaction of VmbHLHs with the large group of PA-type TFs, found to exist in bilberry in our previous study^39^, cannot be ruled out. For example, it is likely that TT8-type VmbHLH2 also interacts with other MYBs since the expression of *VmMYBA1* is restricted to ripening berries^39^. The high expression of the three *VmbHLHs* present in non-fruit bilberry tissues may indicate functions for all of them in both vegetative and reproductive tissues with their role being dependent on the presence of suitable R2R3 MYB partners. Since the *bHLH* expression patterns do not always correlate with *R2R3 MYB* partner expression or product accumulation^22,24,29,68^, the suggested protein-protein interactions between VmbHLHs and their putative 18 R2R3 MYB partners in bilberry needs to be verified in future studies.

ABA treatment did not result in enhanced expression of any of the three *VmbHLHs*, but *VmbHLH1* expression was significantly downregulated and *VmbHLH3* slightly downregulated by ABA. We previously showed that VmMYBA1, VmMYBPA1.1 and VmMYBPA2.2, which are all associated with anthocyanin regulation in bilberry fruit, were upregulated by ABA^39^. This indicated that the bilberry fruit ripening-related anthocyanin biosynthesis induced by ABA is controlled by *MYB* transcription, not the *bHLH* expression. Similar results have been reported previously in strawberries indicating that ABA upregulates the expression of anthocyanin-related *FaMYB1* and *FaMYB10* but does not regulate TT8-type *FabHLH3* while GL3/EGL3-type *FaMYC1* is negatively regulated by ABA^69,70^. The R2R3 MYB is usually considered as the rate-limiting component of the MBW complex, and it can activate expression of *bHLH* and *WD40* genes through a conserved hierarchy^65^. For example, overexpression of Arabidopsis *AtPAP1* was shown to upregulate the expression of *AtTT8*^71^. In black goji berry, LrAN2-like is known to regulate *LrAN1b* expression^29^. Also in our earlier study, VmMYBA1, VmMYBA2, VmMYBPA2.2, and to a certain extent VmMYBPA1.1, were able to activate the expression of TT8-type *NbAN1* in *N. benthamiana* leaves^39^. This hierarchy is supported by our current study, demonstrating that *MdMYB10* could activate *NtAN1* expression in *N. benthamiana* leaves, while *VmbHLHs* could not significantly activate *NtAN2* (MYB) expression. However, based on our study, such hierarchy does not exist in bilberry fruit as elevated *VmMYBA1*, *VmMYBPA1.1* and *VmMYBPA2.2* expression by ABA did not lead to upregulation in TT8-type *VmbHLH2* expression. Since *VmbHLH2* expression increased during bilberry fruit ripening, the induction in expression seems to be regulated by signals or phytohormones other than ABA and needs to be clarified by future studies. As discussed above, hierarchical regulation has also been described to exist between the different IIIf subgroup bHLH members in certain species, commonly MYC1/GL3/EGL3-type bHLHs regulating TT8-type bHLH expression during flowering or fruit ripening^21,29,65,72^. However, it appears that the hierarchical control between bHLH components varies in Eudicots^65^. Thus, this needs to be studied in the future between bilberry bHLHs, and the availability of bilberry genome provides possibilities for promoter activation studies. Similarly, the degree of redundancy among the different IIIf subgroup bHLH members seems to vary by species^72^ and remains to be studied in the genus *Vaccinium* in the future.

## Conclusions

Our study shows, for the first time, the likely alternate functional roles for flavonoid-related bHLH TFs. The evidence suggests that the TT8-type VmbHLH2 is directly involved as the bHLH component in the MBW complex controlling anthocyanin production in berries. Further, the MYC1-type VmbHLH1 and GL3/EGL3-type VmbHLH3 are associated with anthocyanin and PA regulation in unripe fruit and non-fruit tissues.

## Materials and methods

### Bilberry plant material

Bilberry (*Vaccinium myrtillus* L.) fruits at five ripening stages, ranging from unripe green to fully ripe blue (stages 3.0, 3.5, 4.0, 4.5, and 5.0), were collected from Tromsø, Norway (69°75′ N, 19°03′ E). Vegetative bilberry tissue samples were collected from forest stands in Oulu (65°01′ N, 25°28′ E). The formal identification and deposition of voucher specimens into the public herbarium were undertaken by the Department of Arctic and Marine Biology at UiT The Arctic University of Norway. Bilberry fruit treatments with 0.5 mM ABA [(±)-abscisic acid; Sigma-Aldrich, St. Louis, MO, USA], 2 mM ABA or water as negative control were conducted in Petri dishes under sterile conditions as described earlier^35^. Three replicate Petri plates with approximately 50 unripe berries per plate were employed. The plates were kept at 18 °C under 30 µmol m^−2^ s^−1^ light. Berry samples were collected after 48 h from the beginning of the ABA treatment. Immediately after collection, all samples were frozen in liquid nitrogen and stored at − 80 °C before RNA extraction.

### Identification and sequence analysis of bilberry *bHLH* genes

Protein sequences of *Arabidopsis thaliana* bHLHs of subgroup IIIf were used to identify potential bilberry anthocyanin biosynthesis-related *bHLH* genes using blastn searches against bilberry genome representing the Tromsø ecotype^47^, accessible in the Genome Database for *Vaccinium* (https://www.vaccinium.org). Full-length deduced amino acid sequences of bilberry bHLHs were aligned using the Clustal Omega program (https://www.ebi.ac.uk/jdispatcher/msa/clustalo) and visualized using GeneDoc software^73^. For phylogenetic tree analysis, the amino acid sequences of previously characterized plant bHLH subgroup IIIf proteins were obtained from GenBank (Supplementary Table S1). Arabidopsis bHLH sequences were retrieved from UniProt (https://www.uniprot.org/). Full-length protein sequences were aligned with ClustalW and a phylogenetic tree was constructed using the maximum likelihood method with the JTT + G model in MEGA software (v. 12).

### RNA extraction and gene expression analysis in bilberry tissues

Frozen samples were homogenized into a fine powder using a mortar and pestle under liquid nitrogen. Total RNA was isolated from bilberry tissues by using the Spectrum™ Plant Total RNA kit (Sigma-Aldrich) with on-column digestion using DNase I (Sigma-Aldrich). The quality of RNA was verified and quantified using a NanoDrop spectrophotometer (Thermo Fischer Scientific, Waltham, MA, USA) and RNA integrity was verified electrophoretically. Total RNA was converted to cDNA by using the SuperScript™ IV reverse transcriptase (Invitrogen, Carlsbad, CA, USA), according to the manufacturer’s instructions. From ABA-treated berries, total RNA was extracted and cDNA synthesized and purified as described previously^35^.

Real-time quantitative PCR (qPCR) analyses were performed as described previously^74^ using a Bio-Rad CFX96™ Real Time System (Bio-Rad, Hercules, CA, USA) and SsoAdvanced™ Universal SYBR Green Supermix (Bio-Rad). Gene-specific primer sequences used for the qPCR analyses are listed in Supplementary Table S2. All analyses were performed with at least three biological replicates. Relative expression levels of *bHLH* genes were calculated in CFX Maestro™ software (Bio-Rad) using glyceraldehyde-3-phosphate dehydrogenase (*VmGAPDH*) and *VmActin* as reference genes.

### ***VmbHLH*** gene cloning and construction of expression vectors

Full-length coding sequences of three *VmbHLHs* were amplified from bilberry fruit cDNA by PCR using Phusion^®^ High-Fidelity DNA Polymerase (Thermo Fischer Scientific) with gene-specific primers (Supplementary Table S3). The amplified PCR products were ligated into the expression vector pEAQ-*HT*-DEST3 under the control of *CaMV35S* promoter by Gateway cloning. The sequences of the constructed expression vectors were verified by sequencing using the BigDye™ Terminator Cycle Sequencing Kit (Applied Biosystems, Foster City, CA, USA) with a 3130xl Genetic Analyzer (Applied Biosystems) at the UiT The Arctic University of Norway sequencing facility. *MdMYB10* and *MdbHLH3* cloning into a binary expression vector pSAK277 was as previously described in Espley et al.^24^.

### Transient overexpression assays

The expression vectors were transformed into electrocompetent *Agrobacterium tumefaciens* (GV3101) cells, followed by growth on LB agar media supplemented with selective antibiotics at 28 °C. Harvested cells were resuspended in infiltration buffer [10 mM MES (pH 5.6), 10 mM MgCl_2_, 200 µM acetosyringone] to reach an OD_600_ of 0.6. After incubation at room temperature for 2–3 h, the *Agrobacterium* solution was infiltrated into the abaxial side of leaves of 5-weeks-old *Nicotiana benthamiana* using a syringe. *Agrobacterium* cells containing *bHLH*-constructs were introduced to leaves alone or with an equivalent dose of *Agrobacterium* cells containing the *MdMYB10* construct. An empty vector served as a negative control. At least three plants were transformed with each construct combination. Infiltration sites were collected 7 days after the infiltration and stored at − 80 °C until they were used for qPCR analyses and measurement of anthocyanins.

For qPCR analyses, total RNA from the infiltrated sites of *N. benthamiana* leaves was extracted and cDNA prepared as described above. qPCR analyses were performed as described above using gene-specific primers listed in Supplementary Table S4. All analyses were performed with at least three biological replicates. The relative expression levels were calculated in CFX Maestro™ software (Bio-Rad) by using elongation factor 1 (*NbEF1*) and *NbActin* as reference genes.

### Measurement of anthocyanins

Frozen tissues were ground to fine powder with a mortar and pestle under liquid nitrogen. For analyses of anthocyanins from *N. benthamiana* leaves, tissue powder of 0.1 g was extracted and measured spectrophotometrically as described by Chu et al.^75^. All the analyses were performed with three to four biological replicates. The results were expressed as µg (cyanidin-3-glucoside equivalent) g^−1^ fresh weight (FW).

### Statistical analysis

The quantitative results of gene expression and anthocyanin determination were analyzed with Student’s *t*-test using SPSS Statistics program, version 29.0.2.0 (IBM, New York, NY, USA).

## Supplementary Information

Below is the link to the electronic supplementary material.

Supplementary Material 1

## Data Availability

The data used to support the findings of this study are included within the manuscript and its supplementary information files.
