# Supplementary material for: Three bilberry bHLHs of subgroup IIIf play divergent roles in fruit anthocyanin and flavonoid biosynthesis
Source: Sci Rep. 2025 Aug 14;15:29816. doi: 10.1038/s41598-025-15557-w (PMC12354701; doi:10.1038/s41598-025-15557-w)
Supplement: Supplementary file 1 — Supplementary Material 1 [file 41598_2025_15557_MOESM1_ESM.pdf]

Three bilberry bHLHs of subgroup IIIf play divergent roles in fruit anthocyanin and flavonoid biosynthesis

Katja Karppinen[1,*], Lauri Raami[1,2], Hilary Edema[1], Muhammad Furqan Ashraf[1], Heikki M. Salo[2], Richard V. Espley[3] & Laura Jaakola[1,4]

[1] Department of Arctic and Marine Biology, UiT The Arctic University of Norway, Tromsø, Norway

[2] Ecology and Genetics Research Unit, University of Oulu, Oulu, Finland

[3] Plant and Food Research Group, Bioeconomy Science Institute, Auckland, New Zealand

[4] Division of Food Production and Society, Norwegian Institute of Bioeconomy Research (NIBIO), Ås, Norway

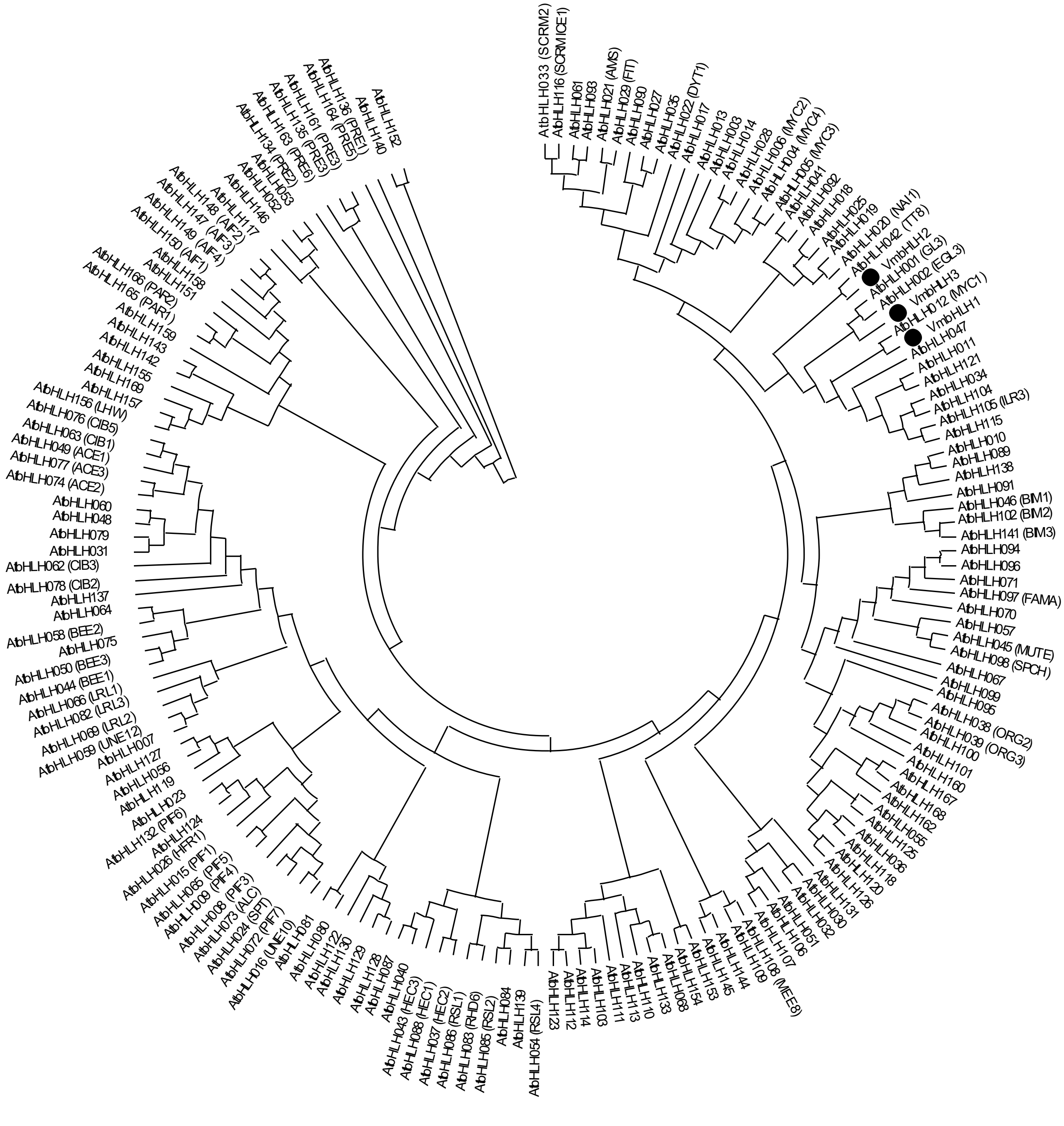

Figure S1. Phylogenetic analysis of VmbHLHs with 169 Arabidopsis AtbHLHs. The three bilberry bHLHs are indicated as black circles. The tree was constructed using the maximum likelihood method in MEGA 12 with 500 bootstrap estimates.

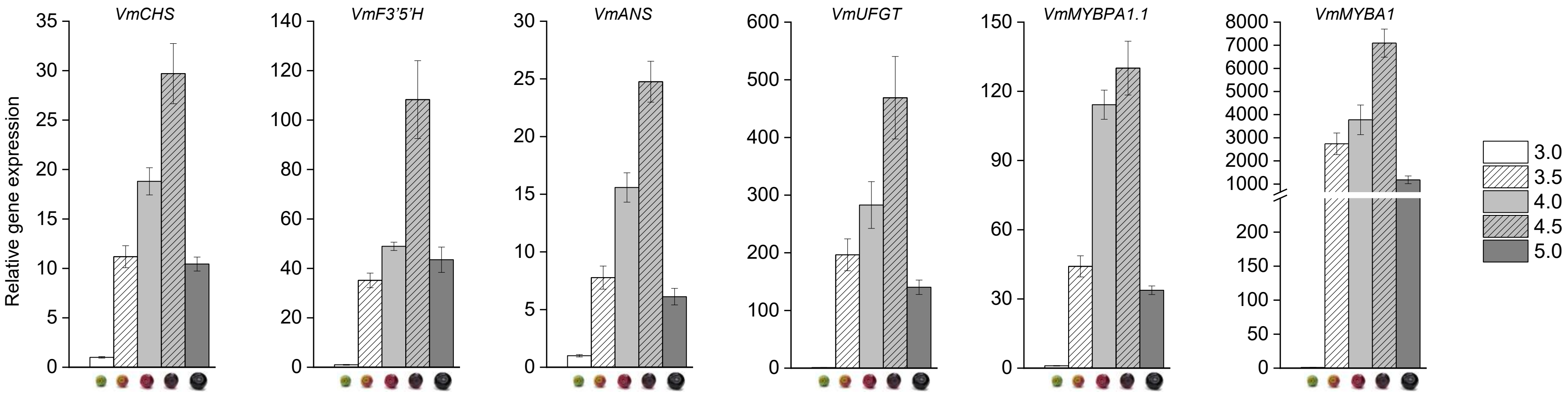

Figure S2. The expression profiles of key fruit anthocyanin biosynthetic genes and main R2R3 MYB regulators during bilberry fruit ripening quantified by qPCR. Values represent means ± SEs of four replicates. 3.0-5.0 represent fruit ripening stages from unripe to fully ripe berry.

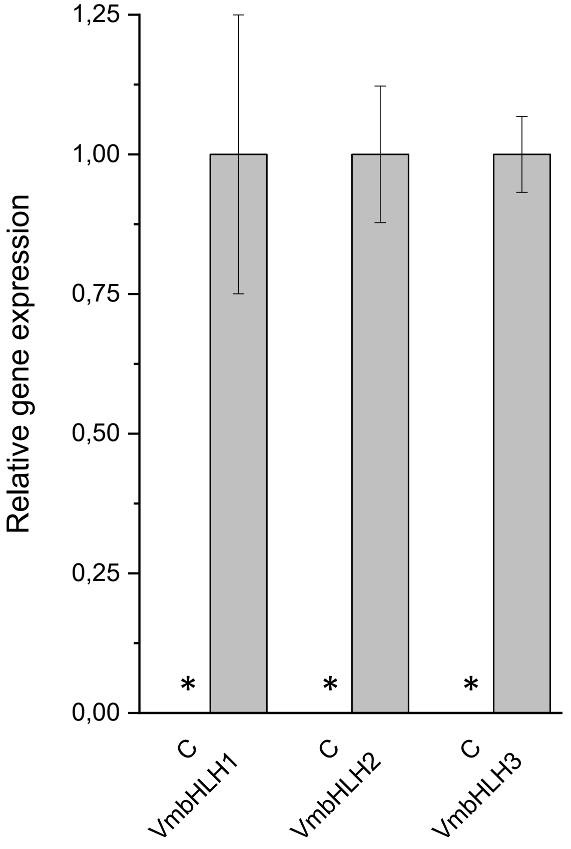

Figure S3. Transcript levels of *VmbHLHs* after overexpression in *Nicotiana benthamiana* leaves. Relative expression was quantified from infiltration sites after 7 days of infiltration by qPCR. Expression levels are presented for leaves infiltrated with *VmbHLH* construct together with *MdMYB10*. The asterisks (*) represent no detection of gene expression in negative controls (C) infiltrated with empty vector. Values represent means ± SEs of at least three biological replicates.

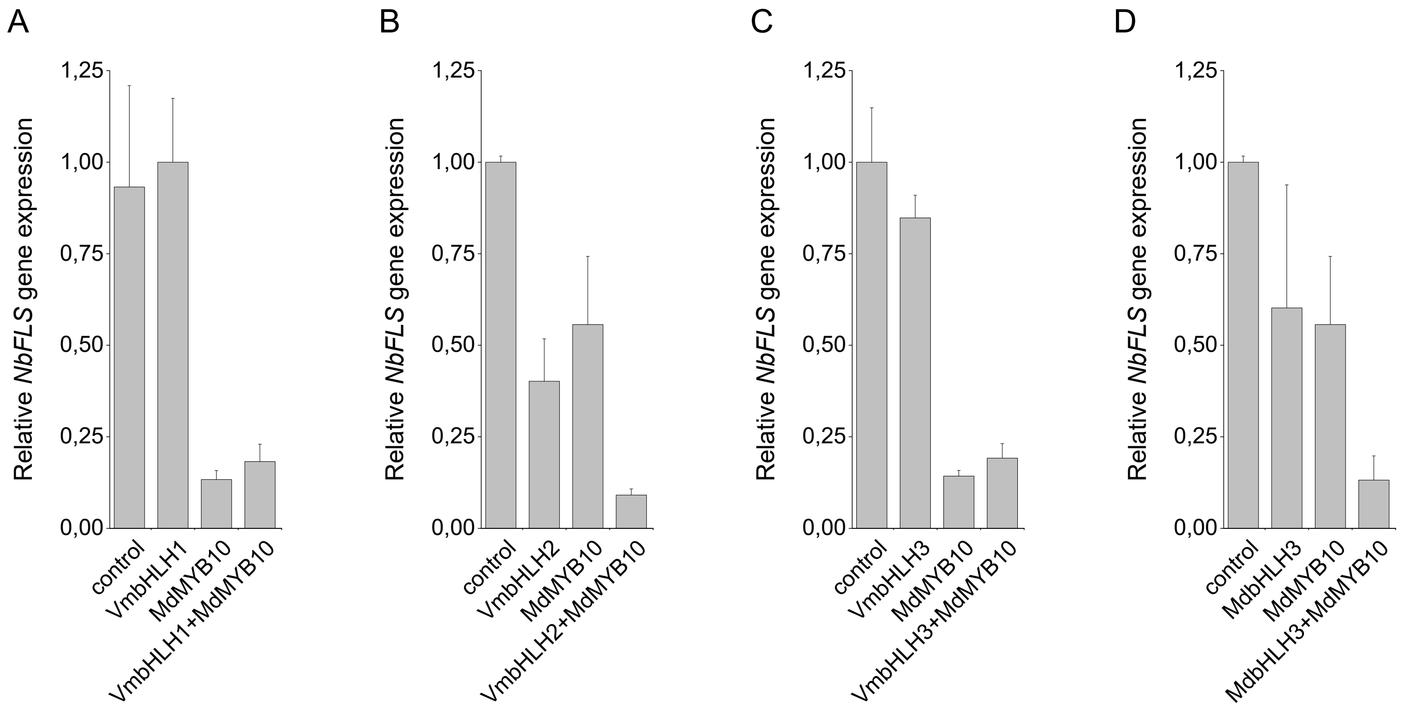

Figure S4. Expression of *NbFLS* gene in *Nicotiana benthamiana* leaves after transient overexpression of (A) *VmbHLH1*, (B) *VmbHLH2,* (C) *VmbHLH3* and (D) *MdbHLH3* (positive control). The relative gene expression was quantified from infiltration sites after 7 d of infiltration by qPCR and normalized to *NbEF1* and *NbActin*. Values represent means ± SEs of at least three biological replicates.

Table S1. Subgroup IIIf of bHLH protein sequences utilized for phylogenetic analysis.

Species	bHLH protein	Accession ID
Antirrhinum majus	AmDELILA	AAA32663
Arabidopsis thaliana	AtbHLH001 (GL3)	At5g41315
	AtbHLH002 (EGL3)	At1g63650
	AtbHLH012 (MYC1)	At4g00480
	AtbHLH042 (TT8)	At4g09820
Brassica rapa	BrTT8	AEA03281
Citrus sinensis	CsMYC2	ABR68793
Fragaria × ananassa	FabHLH3	AFL02463
Gerbera hybrida	GhyMYC1	CAA07615
Gossypium hirsutum	GhDEL65	AAK19613
Lilium hybrid	LhbHLH2	BAE20058
Malus × domestica	MdbHLH3	ADL36597
	MdbHLH33	ABB84474
Morella rubra	MrbHLH1	AGO58372
	MrbHLH2	AGO58373
Nicotiana tabacum	NtAN1a	AEE99257
	NtAN1b	AEE99258
Perilla frutescens	PfMYC-RP	BAA75513
Petunia × hybrida	PhAN1	AAG25927
	PhJAF13	AAC39455
Prunus persica	PpbHLH3	AIE57508
Vaccinium corymbosum	VcbHLH1-1	VaccDscaff28-augustus-gene-45.27
	VcbHLH1-2	VaccDscaff44-augustus-gene-0.19
	VcbHLH2	VaccDscaff19-augustus-gene-381.30
	VcbHLH004	AOY34375.1
Vitis vinifera	VvMYCA1	NP_001267954
	VvMYC1	ACC68685

Table S2. Bilberry gene-specific primers used for qPCR analyses.

Gene	Forward primer sequence 5′-3′	Reverse primer sequence 5′-3′
VmCHS	CATGATGTACCAACAGGGTTGC	GTGATCTCAGAGCAAACCACCA
VmF3'5'H	GATTGCGTGGATGGACTTACA	AAATCTGGGTTCCCTTTACGC
VmANS	GCAACTCTTCTACGAGGGCAAA	CCTGTGGAGAATGCTCTTGCAC
VmUFGT	CATCCAAACCCTGTTCCCATCC	TCATCCCTGCCTTCAAGCTCTC
VmMYBPA1.1	GGACATTCAACGCCAATCTGGT	CGGCAAAGGAATCCAACTGAAG
VmMYBA1	CTCGACCACAAACCTTGTCCA	GCCTCCTCATTTGATCCGTCA
VmbHLH1	TAGCGGAGCATGAAGCTAGAAG	ACAGTGGCCCATCTTTAGGAAC
VmbHLH2	CAGGTGCAGGTGTCGATTATAG	CCGTTGTCTTTCACCTTAGCTC
VmbHLH3	GCCTCATTGTCCATGCTAAACC	GCTGAGGATTTATCCCGAAAGC
VmActin	TTCCCTGGGATTGCTGATAG	GGTCTTGGCAATCCACATCT
VmGAPDH	GCTGTACCACAAACTGTCTTGC	ATGAAGCAGCTCTTCCACCTCT

Table S3. Primers used for construction of *VmbHLH* expression vectors for transient overexpression.

Gene	Primer sequence 5′-3′
VmbHLH1-forward	GGGGACAAGTTTGTACAAAAAAGCAGGCTATGGCTAAAGGGGCACAAAACCAA
VmbHLH1-reverse	GGGGACCACTTTGTACAAGAAAGCTGGGTAACACTTTCCGGCAACAGTTGAAAG
VmbHLH2-forward	GGGGACAAGTTTGTACAAAAAAGCAGGCTATGGCCGCACCACCTAGTACACGGC
VmbHLH2-reverse	GGGGACCACTTTGTACAAGAAAGCTGGGTAGTACGAGGGTATGATTTGGTGTATTG
VmbHLH3-forward	GGGGACAAGTTTGTACAAAAAAGCAGGCTATGGCTTCTTTGCCAGAAGACCTC
VmbHLH3-reverse	GGGGACCACTTTGTACAAGAAAGCTGGGTTACTCTTCCGAACGATTCTCTGAATC

Table S4. *Nicotiana benthamiana* gene-specific primers used for qPCR analyses.

Gene	Forward primer sequence 5′-3′	Reverse primer sequence 5′-3′
NbCHS	CCAATGACACCCACTTGGATAG	CAGAGACAAGCTGGAACAAAGG
NbF3H	AGGTGGCTTCATTGTCTCTAGC	TCACAGCTATCCATCCTTCTGG
NbF3'H	CGTCCTCAAGCACAGTAGAATG	CTAGGTCAGATTCCATCACGAG
NbF3'5'H	CTCGAACGAACCATGTGAAGTG	TCCCACTCAAGAACCTTTCAGG
NbDFR	GGCAACACCTATGGATTTCGAG	TCGAGAGTTCCAGCAGATGAAG
NbANS	AGCAGATCAGGAACCTAGCAAC	CAGTATGAGCTTCGACACCAAG
NbUFGT	GGACTGCTGGTTCTGGTTCTTT	GCTACAACTTCTGGTGGCATGT
NbLAR	ATGAAGTGGACAGGTCAGATCC	AATGGTCAAGAGGAGGAGGAAG
NbANR	GCAGAGCAAATGTATCGTCCAG	GGGCATGCAGATGTTATCAGGT
NbFLS	TCACTTGGGCTTGGGTTAGAAG	ATGTCTGTATGGGCCACAACTC
NbAN1	CATCTCTTAATAATGGCGTCTTCTTG	CTCTAGGGATTATCTGATGTATTGACC
NbAN2	GGAAAAGTTGCAGACTGAGGTG	ACCCGCAATAAGTGACCATCTG
NbEF1	AACCCTTCTTGAGGCTCTTGAC	GTCCAAAGGTCACAACCATACC
NbActin	GAGCGGGAAATTGTTAGGGATG	AGCGGAATCTCTCAGCACCAAT
